# Obstructive Sleep Apnea Phenotypes and Markers of Vascular Disease: A Review

**DOI:** 10.3389/fneur.2017.00659

**Published:** 2017-12-05

**Authors:** Alberto R. Ramos, Pedro Figueredo, Shirin Shafazand, Alejandro D. Chediak, Alexandre R. Abreu, Salim I. Dib, Carlos Torre, Douglas M. Wallace

**Affiliations:** ^1^Department of Neurology, Miller School of Medicine, University of Miami, Miami, FL, United States; ^2^Sleep Disorders Center, Miller School of Medicine, Bascom Palmer Eye Institute, University of Miami, Miami, FL, United States; ^3^Department of Medicine, Miller School of Medicine, University of Miami, Miami, FL, United States; ^4^Department of Otolaryngology – Head and Neck Surgery, Miller School of Medicine, University of Miami, Miami, FL, United States; ^5^Sleep Disorders Center, Bruce W. Carter VA Medical Center, Miami, FL, United States

**Keywords:** obstructive sleep apnea, phenotype, stroke, cardiovascular disease, sleep disordered breathing

## Abstract

Obstructive sleep apnea (OSA) is a chronic and heterogeneous disorder that leads to early mortality, stroke, and cardiovascular disease (CVD). OSA is defined by the apnea–hypopnea index, which is an index of OSA severity that combines apneas (pauses in breathing) and hypopneas (partial obstructions in breathing) associated with hypoxemia. Yet, other sleep metrics (i.e., oxygen nadir, arousal frequency), along with clinical symptoms and molecular markers could be better predictors of stroke and CVD outcomes in OSA. The recent focus on personalized medical care introduces the possibility of a unique approach to the treatment of OSA based on its phenotypes, defined by pathophysiological mechanisms and/or clinical presentation. We summarized what is known about OSA and its phenotypes, and review the literature on factors or intermediate markers that could increase stroke risk and CVD in patients with OSA. The OSA phenotypes where divided across three different domains (1) clinical symptoms (i.e., daytime sleepiness), (2) genetic/molecular markers, and (3) experimental data-driven approach (e.g., cluster analysis). Finally, we further highlight gaps in the literature framing a research agenda.

## Introduction

Obstructive sleep apnea (OSA) is a chronic disorder that leads to early mortality, stroke, and cardiovascular disease (CVD) (1–5). OSA affects 34% of men and 17% of women of all ages, with an estimated cost of $149.6 billion in 2015 from untreated OSA (6–9). Obstruction of the upper airway during sleep causes hypoxemia, sleep fragmentation, and sympathetic nervous system activation (6–8). OSA may lead to stroke through its associations with potent vascular risk factors, such as hypertension, diabetes mellitus, obesity, and atrial fibrillation. OSA may also increase stroke and CVD risk through reduction in cerebral blood flow, altered cerebral autoregulation, impaired endothelial function, increased platelet activation, inflammation, and oxidative stress related to the intermittent hypoxemia–reoxygenation and arousals associated to increased sympathetic tone (10). The treatment of OSA has been considered an important intervention for reducing the morbidity and mortality associated with stroke and CVD (11). However, treatment of OSA has not consistently reduced cardiovascular risk (12, 13); results partly explained by methodological limitations, such as suboptimal adherence to positive airway pressure therapy. Importantly, some limitations may lie on the need to better identify the subset of individuals who respond more favorably to OSA therapy, with the potential of a greater reduction in adverse outcomes, for further review consider the article by Drager et al. (14). Recently, the emphasis on personalized medical care introduces the possibility of a unique approach to the treatment of OSA based on its pathophysiological mechanisms and/or clinical presentation (15, 16). Personalized medicine can lead to targeted clinical and public health strategies for reducing stroke and CVD risks associated with OSA.

Historically, OSA has been considered a uniform condition defined by an apnea-hypopnea index (AHI) ≥5 events per hour of sleep (17). The AHI combines apneas and hypopneas associated with hypoxemia. Yet, other sleep metrics (i.e., oxygen nadir, arousal frequency), along with clinical symptoms and molecular markers could be better predictors of stroke and CVD outcomes in OSA (17–19). The heterogeneity of clinical presentations leads to the evaluation of OSA phenotypes as a strategy to identify at-risk individuals for stroke and CVD (18, 19). As defined in the article by Zinchuk et al., a phenotype is “a category of patients with OSA distinguished from others by a single or combination of disease features, in relation to clinically meaningful attributes (symptoms, response to therapy, health outcomes, quality of life)” (20). The authors further define *clinical phenotypes* based on *a priori* categories according to the presence or absence of symptoms (e.g., daytime sleepiness). *Molecular-genetic* phenotypes are based on molecular features (i.e., DNA and RNA). Empirical or data-driven phenotypes are based on statistical analyses (e.g., cluster) examining a multitude of sleep symptoms, demographics, clinical, and physiological data to define distinct OSA subtypes (20).

The high prevalence of undiagnosed OSA and its resultant stroke and CVD risk provide a strong impetus to develop better risk-stratification strategies (18, 19). There is a need to further define the OSA phenotypes that may differentially affect stroke and or CVD risk. Thus, the focus of this systematic review is to summarize what is known about OSA and its phenotypes, in relation to the intermediate markers that increase stroke and CVD risk in OSA. We further highlight gaps in the literature that frame a research agenda.

## Methods

We performed a systematic review by searching the PubMed and Embase databases using the PRISMA guidelines (21). The search strategy was constructed using MeSH terms. The initial search was conducted with the terms “obstructive sleep apnea” or “sleep disordered breathing” and “phenotype” or “phenotypes.” Also articles were searched with the key word “obstructive sleep apnea phenotypes.” The first author evaluated the abstract for articles that included “cardiovascular disease,” or “stroke,” or “cerebrovascular disease.” The search included articles written from January 1, 2007 until November 1, 2017.

To be included in the review, articles had to be primary research studies, published in peer-reviewed journals, written in English and included adults (≥18 years old). Articles were excluded if they were (1) animal studies; (2) a pediatric population; (3) written in languages other than English; (4) conference abstracts; (5) case reports or case series; and (6) commentary or review articles.

A total of 153 articles were retrieved and the abstract were reviewed by the lead author for inclusion and exclusion criteria. A total of 14 articles met the inclusion criteria and were independently reviewed for additional information by the authors (Alberto R. Ramos, Pedro Figueredo, and Douglas M. Wallace). The lead author conducted ancestry search of all retrieved articles’ reference lists. The authors (Alberto R. Ramos, Douglas M. Wallace) also searched the major sleep journals including Sleep Medicine Reviews, Sleep, the Journal of Clinical Sleep Medicine, Sleep Medicine, CHEST, Thorax, and Sleep and Breathing. This resulted in two additional articles. Article tracking software (COVIDENCE) was used to manage the retrieved literature.

## Results

The majority of articles used clinical symptoms (*n* = 8) to define OSA phenotypes followed by experimental or data-driven approach (*n* = 5) and by molecular-genetic studies (*n* = 4). Most of the articles were cross-sectional analyses, case–control, or retrospective studies from sleep disorder centers (22–29). Two studies, Patel et al. (30) and Luyster et al. (23) were from community or population-based samples. The study from, Saaresranta et al. (28) and Zinchuk et al. (31) were longitudinal studies. Most studies comprised male participants, except for the two population based studies, Patel et al. (30) and Luyster et al. (23), and Masa et al. (32), which had 55 and 65, and 65% of females, respectively (Table 1).

**Table 1:** Reviewed studies with obstructive sleep apnea phenotype and vascular markers.

Study	Design/setting	Sample/age (years)/sex	Control/reference group	Study interval	Phenotype definition/outcome(s)
Andaku et al. (33)	Case–control Sleep laboratory	*N* = 35	AHI < 5	–	Clinical/inflammatory markers
		42–45 ± 9.46–10.56			
		100% males			

Bailly et al. (26)	Cross-sectional Sleep laboratory	*N* = 18,263	–	–	Experimental/cardiovascular disease (CVD)
		Age 59 (50–67)			
		73.8% males			

Chen et al. (27)	Case–control Sleep laboratory	*N* = 24	AHI < 5	2012–2014	Clinical-molecular/hypertension
		51.8 ± 8.9			
		86.7% males			

Chen et al. (34)	Cross-sectional Sleep laboratory	*N* = 116	AHI < 5	2012–2014	Molecular/excessive daytime sleepiness
		Age range 20–65 years			
		75% males			

Joosten et al. (22)	Retrospective Sleep laboratory	*N* = 1064	–	2007–2009	Experimental/BMI
		50.9 ± 13.0			
		66% males			

Koyama et al. (35)	Cross-sectional sleep laboratory	*N* = 266	–	–	Molecular genetic/hypertension
		48 ± 13			
		100% males			

Li et al. (36)	Cross-sectional Sleep laboratory	*N* = 58	–	–	Clinical/inflammatory markers
		53.7 ± 7.0			
		63.8% males			

Luyster et al. (23)	Cross-sectional community cohort	*N* = 519	AHI < 5	–	Clinical/lipoprotein
		58.7 ± 7.4			
		65% females			

Masa et al. (32)	Cross-sectional Tertiary hospital	*N* = 302	–	2009–2013	Clinical/CVD
		61.7 ± 12.3			
		61.9% females			

Palma et al. (24)	Case–control sleep laboratory	*N* = 164	AHI < 5	2012–2013	Clinical/vascular autonomic function
		49.1–54.3 ± 9.8–12.2			
		7–18.2%			

Patel et al. (30)	Cross-sectional/community cohort	*N* = 972	AHI < 5	–	Molecular-genetic/hypertension
		45.6 ± 15.8			
		55.3% females			

Saaresranta et al. (28)	Longitudinal sleep laboratory	*N* = 6,555	–	2007–2012	Clinical/CVD
		Age range 50–55 years			
		75% males			

Vavougios et al. (29)	Retrospective/sleep laboratory	*N* = 1,472	–	2011–2013	Experimental/Charlson comorbidity index
		49.4 ± 6.1			
		83.9% males			

Wang et al. (25)	Retrospective sleep laboratory	*N* = 508	AHI < 5	2009–2012	Clinical/hypertension
		50.0 ± 13.0			
		76% males			

Ye et al. (37)	Cross-sectional sleep laboratory	*N* = 822	–	–	Experimental/vascular risk
		54.5 ± 10.6			
		81% males			

Zinchuk et al. (31)	Longitudinal DREAM study	*N* = 1,247	AHI < 5	2000–2012	Experimental/death, CVD
		Age 58.3 ± 11.7			
		94.9% males			

*AHI, apnea–hypopnea index; DREAM, Determining Risk of Vascular Events by Apnea Monitoring*.

### Clinically Defined Phenotypes

The most common clinical phenotype (OSA subtype) was the patient with OSA and excessive sleepiness (EDS). In most studies, the patient with OSA and EDS had increased hypertension, inflammatory markers, and cardiovascular comorbidities (33). For example, the study from Andaku et al. (33) evaluated patients with metabolic syndrome and OSA. The authors compared patients with and without subjective daytime sleepiness. These were also compared to a control group with metabolic syndrome but without OSA and without daytime sleepiness. The authors described higher inflammatory markers (e.g., CRP) in participants with OSA and daytime sleepiness compared to the other groups adjusting for waist circumference, HOMA-IR, and triglycerides, without differences in oxidative stress markers. In a different cohort (36), 58 OSA patients (67% males), had measures of subjective sleepiness (using Epworth sleepiness scale and Stanford Sleepiness scale), and objective sleepiness with four consecutive nights of polysomnography followed by multiple sleep latency test (MSLT). In this sample, lower MSLT scores were associated with increased night time and daytime levels of interleukin-6, while subjective sleepiness was not associated with increased inflammation. In contrast, the largest cohort (*n* = 6,555) of OSA patients (28) divided participants into four groups based on the presence of insomnia (yes vs no) and daytime sleepiness (yes vs no). Different to other studies, daytime sleepiness was not associated to excess CVD risk.

Of interest, the study by Masa et al. (32), evaluated a 302 patients with OSA and comorbid obesity hypoventilation syndrome. The OSA phenotypes were defined by tertiles of oxygen desaturation index. Patients in the highest tertile, had increased daytime sleepiness, obesity, mostly males and more symptomatic. Paradoxically, the highest tertile had decreased cardiovascular comorbidities, such as stroke, arrhythmia, and pulmonary hypertension, among others.

### Molecular-Genetic Studies

The four studies reviewed, evaluated the genetic and epigenetic markers associated with OSA clinical phenotypes and hypertension (27, 30, 34). Chen et al. (27) observed that alteration of the natriuretic peptide receptor 2 and its pathways was associated with the clinical phenotype of daytime sleepiness in OSA patients. In a different study from Chen et al. (34), decreased expression of BIRC3 gene was associated with the development of hypertension in OSA patients. In a relatively large sample (*N* = 972) of the Cleveland Family Study, Patel et al. (30) showed that insertion (I)/deletion (D) polymorphisms of the angiotensin converting enzyme were associated with hypertension in OSA patients with AHI > 30. Of interest, participants with a DD genotype had 37% reduction in the odds of hypertension compared to II genotype. However, Koyama et al. (35) found that among those with hypertension the II genotype was inversely associated with OSA severity [OR 0.27 (95% CI 0.09–0.80); *p* = 0.017] (Table 2).

**Table 2:** Genetic and molecular markers associated to hypertension in patients with obstructive sleep apnea.

	Molecular marker	Outcome	Statistical measure
Chen et al. (34)	BIRC3 protein expression	Hypertension	0.04 ± 0.06 pg/ml (hypertension) vs. 0.21 ± 0.33 pg/ml (no hypertension), *p* = 0.048

Koyama et al. (35)	II genotype compared to ID + DD genotype	Hypertension	OR 0.27 (95% CI 0.09–0.80)

Patel et al. (30)	DD genotype vs. others	Hypertension	OR 0.57 (95% CI 0.26–1.24)

### Experimental-Analytical

Experimental-analytical (data driven) studies suggest a heterogeneity of clinical phenotypes (22, 29, 37). The Icelandic Sleep Apnea Cohort (*n* = 822) of patients with moderate to severe sleep apnea (AHI ≥ 15) described three different phenotypic clusters. Cluster 1 had predominantly insomnia symptoms, cluster 2 had minimal symptoms compared to the other clusters, and cluster 3 was characterized by excessive daytime sleepiness (EDS). Interestingly, 42% of patients were in cluster 3, hence the majority of patients did not report EDS. Also patients from cluster 2 had the highest odds of hypertension (OR = 1.38 *p* ≤ 0.001) and CVD (OR = 1.67; *p* < 0.001) compared to cluster 3. These clusters were not clinically different in age, sex, BMI, AHI, or oxygen desaturations (37). A different, cluster analysis identified six phenotypic clusters associated with a number of medical and psychiatric comorbidities. The highest amount of vascular comorbidities was found in the middle-aged OSA patient with multiple sleep complaints (e.g., snoring, apnea, sleepiness) and in the obese-older patient with minimal sleep symptoms (29).

An analysis of a large sample from the French National registry of sleep apnea (*N* = 18, 263) included participants with moderate to severe sleep apnea (AHI ≥ 15), mean age of 59 years and predominance of males (73.8%). A hierarchical cluster analysis identified six clusters that varied by age, sleep symptoms, and medical comorbidities. Two different clusters had increased prevalence of stroke, cardiac arrhythmias, hypertension, and diabetes mellitus. Patients from cluster 3 were older patients (mean age 66) with less daytime sleepiness, while patients from cluster 6 were symptomatic middle-aged patients (mean age 60) with higher levels of sleepiness. These two clusters had the highest AHI and worse oxygen desaturations when compared to the other clusters (26). A longitudinal analysis of the 1,247 participants of the multi-center DREAM study, Determining Risk of Vascular Events by Apnea monitoring, used *K*-means analysis to create clusters based on polysomnography variables. The authors also used Cox proportional hazards models to evaluate the clusters associated with the composite outcome of acute coronary syndrome, transient ischemic attack, stroke, or death. The authors identified seven clusters. Most of them had increased risk of adverse cardiovascular events. However, the two clusters with the highest risk were labeled the “PLMS” cluster and the “arousal and poor sleep” cluster (31). The “PLMS” cluster had higher periodic limb movements in sleep with relatively mild AHI. This cluster had a hazard ratio (HR) of 2.36 [95% confidence interval (CI) of 1.61–3.46] of the composite outcome. The “Arousal and poor sleep” cluster was characterized by severe AHI, without hypoxic burden, but predominantly poor sleep architecture and many arousals. These cluster was associated with lower use of positive airway pressure and increase prevalence vascular risk factors. This cluster had a HR of 2.33 with 95% CI of 1.32–4.10. Of interest, sleep apnea was not associated with adverse cardiovascular events when using the AHI cutoffs of mild (<15), moderate (15 ≤ 30) and severe (≥30).

## Discussion

The purpose of this review was to systematically evaluate manuscripts with concurrent definition of sleep apnea phenotypes and measures of vascular disease. As previously described, *a priori* symptom classification showed that the OSA-excessively sleepiness (EDS) phenotype, had increased mortality and adverse vascular outcomes. Interestingly, the OSA-EDS phenotype was associated with increased inflammatory markers, such as CRP and interleukin-6. Similarly, increased inflammation is associated with atherogenesis, hence increased inflammation could mediate the association between OSA-EDS phenotype and atherosclerotic disease. Epidemiological studies show that EDS is observed in up to 50% of patients from sleep centers; therefore, many patients do not endorse EDS. While these is not fully understood, the study from Chen et al. (27), suggest that hypomethylation of genes involved in the natriuretic peptide receptor-2 pathways could lead to EDS in OSA (27). These findings, coupled with the increased in CVD among the OSA-EDS phenotype, suggest a genetic susceptibility to the effects of hypoxia (26). Interestingly, the study from Masa et al. (32) suggests that severe hypoxemia could be protective at least in certain populations (obesity hypoventilation). A possible explanation is ischemic precondition, an adaptive mechanism where low levels of hypoxemia/ischemia leads to vascular and endothelial changes that “protects” against worse vascular events and possibly explaining the lower mortality rates of older age groups with OSA (38).

Interestingly, studies using empirically or data-driven analytical methods such as cluster analysis, observed increased cardiovascular outcomes in patients without daytime sleepiness, but rather clusters that were defined as having “poor,” “fragmented” sleep predominantly with insomnia symptoms or periodic limb movements had increased cardiovascular outcomes. Using a data-driven approach provide an avenue to further identify at at-risk individuals that otherwise may not be treated using AHI cutoffs. As noted in the study by Zinchuk et al. (31), the OSA using the AHI cuttoffs did not predict cardiovascular outcomes. This is consistent with other studies were the AHI did not correlate strongly with most relevant OSA comorbidities, such as hypertension (39). However, as noted in these studies (22, 26, 29, 37), the “classic” OSA patient (snoring, obese, daytime sleepiness) is not the most common phenotype.

In our systematic review, most studies sampled middle-aged males from sleep centers. Of importance, studies derived solely from sleep centers are of limited external validity and generalizability due to possible selection bias. Consequently, there is a need to develop tools for health-care professionals, sleep specialist, and scientist to further recognize and risk-stratify different OSA phenotypes; particularly in older-adults and females (40, 41). Importantly, population-based studies can help further evaluate age, sex and ethnic differences in OSA phenotypes; allowing clinicians and researchers to recognize participants an increased risk of stroke and CVD (40, 41), especially in populations with large burden of CVD that may benefit from inclusion in treatment studies.

Most of studies did not measured the physiologic OSA phenotypes or endotypes, as described by Eckert (42). An anatomical predisposition for upper airway collapse is necessary for OSA; however, these phenotypic classification describe non-anatomical pathways to upper airway obstruction, such a low respiratory arousal threshold, and instability of ventilator control causing an increase in “loop gain.” Of interest, a case–control study compared differences in cardiac autonomic tone in three different groups, OSA patients with hypoxia, OSA without hypoxia and controls (AHI < 5) (24). The authors observed an increased sympathetic contribution in patients with OSA and hypoxia. As noted by the authors, patients with increased loop gain can have partial airway obstruction leading to “apneas” without hypoxia. In contrast, patients with decreased loop gain coupled with factors that worsens upper airway collapse, could increase the length of apneas and arousal thresholds leading to periods of hypoxia.

## Limitations

We may have not included all studies describing OSA phenotypes. We only considered studies with OSA phenotypes, which also had information on risk factors, vascular measures or comorbidities (e.g., hypertension, diabetes) known to increase the risk of stroke and CVD. In addition, articles describing OSA phenotypes, but did not label the study as such, may have not being included in this review as part of our search strategy.

## Future Directions

This review provides a framework to further understand clinical, molecular and genetic information using data-driven approaches to reveal OSA subtypes. There are several important clinical phenotypes that have not been studied comprehensively. For example, the clinical presentation of OSA in pre- and post-menopausal women has been described to be significantly different than that of men (18). Similarly, race–ethnic differences may exist for OSA but have yet to be explored (43). Novel methods for diagnosis of sleep apnea (home sleep test) and use of commercially available wearables could provide further venues to define and identify patients at risk. In addition, phenotyping body position and sleep architecture needs to be incorporated into the current paradigms, as this may differently affect vascular disease and provide different intervention strategies such as positional therapy (22). There is a paucity of longitudinal studies examining whether OSA subtypes are stable over time. In addition, there are no studies determining whether OSA subtypes respond differentially to OSA treatments. Future studies should consider different OSA phenotypes with the intent to evaluate them against clinical outcomes, especially to optimize the use of clinical trials (14). Determining and evaluating the OSA phenotypes at risk for stroke or CVD can provide the structure for clinical trials in the treatment of OSA and reduction of CVD morbidity and mortality. Importantly, novel genetic and molecular markers are necessary. For example, micro-RNAs have been associated to increased atherosclerosis and CVD and recently have been shown to cause endothelial dysfunction associated to the intermittent hypoxia of patients with OSA (44). Future efforts should consider analyzing large-scale cohorts, in collaboration with geneticist and use of expertise in informatics and big data science.

## Author Contributions

Drafting/revising the manuscript for content, including medical writing for content, study concept or design, analysis and interpretation of data.

## Conflict of Interest Statement

The authors declare that the research was conducted in the absence of any commercial or financial relationships that could be construed as a potential conflict of interest.
